# Prevalence and Patterns of Dyslipidemia Among Patients With Rheumatoid Arthritis: A Retrospective Cohort Study

**DOI:** 10.7759/cureus.99682

**Published:** 2025-12-20

**Authors:** Muna M Aldhuhoori, Ibtehal Makki, Maryam Alnuaimi, Fatima Lootah

**Affiliations:** 1 Family Medicine, Graduate Medical Education, Mohammed Bin Rashid University of Medicine and Health Sciences, Dubai Health, Dubai, ARE

**Keywords:** antibody, autoantibody, autoimmune, cardiac, cardiovascular risk, dyslipidemia, early detection, healthy lifestyle, retrospective study, rheumatoid arthritis

## Abstract

Background: Dyslipidemia is a major comorbidity among rheumatoid arthritis (RA), leading to an increased risk of cardiovascular diseases (CVD). Despite its clinical significance, the prevalence and patterns of dyslipidemia in RA are understudied, specifically in diverse populations. Understanding these patterns is essential for developing specific interventions to reduce cardiovascular risk in this population.

Objective: The objective of this study is to assess the prevalence and patterns of dyslipidemia among patients with RA in Dubai, examining lipid profile changes before, at, and after diagnosis and exploring associated demographic and laboratory factors that may contribute to cardiovascular risk.

Methods: A retrospective cohort study was conducted using data from the Epic medical record system in the Rheumatology Department of Dubai Hospital, Dubai Health, Dubai, UAE, between January 2019 and December 2020, with two-year time frames. One hundred and four patients were included in the study. All were adults aged 21-65, and all their data demographics and laboratory variables were collected from the medical record.

Results: The sample was predominantly female, middle-aged, obese, and Emirati. The prevalence of dyslipidemia among RA patients was 78 (75%) before RA diagnosis. Among those with dyslipidemia, elevated low-density lipoprotein (LDL) levels were the most common abnormality (55, 52.9%), followed by reduced high-density lipoprotein (HDL) levels (41, 39.4%). At the time of RA diagnosis, 76 (73.1%) had dyslipidemia, with 52 (50%) having predominantly low HDL levels. After RA diagnosis, 72 (69.2%) had dyslipidemia, with 44 (42.3%) having predominantly high LDL levels. Significant lipid abnormalities were observed in all three phases of the disease during the study. No significant association was found between dyslipidemia and demographic characteristics.

Conclusion: This study showed the prevalence of dyslipidemia among RA patients in Dubai, highlighting the importance of monitoring lipid profiles in RA patients and effective treatment to reduce the risk of CVD in the population for better outcomes.

## Introduction

Rheumatoid arthritis (RA) is the most common chronic autoimmune disease, which involves chronic inflammation. It primarily affects small- to medium-sized joints, causing bone erosions, destruction of the joint, and eventually loss of joint function. It can also impact other systems in the body, such as the cardiovascular, respiratory, and hematologic systems, which could be more commonly anticipated in chronic cases or cases without adequate treatment. Autoimmune thyroid disease, particularly hypothyroidism, has also been reported to occur more frequently among patients with RA, which may further influence lipid metabolism [1].

The disease affects 1% of the population globally, with a prevalence of 460 cases per 100000 people. It was reported that North Africa and the Middle East region combined account for 13% of the global cases of RA, although detailed country-specific data from the Middle East remain limited. The exact etiology remains unknown; however, some risk factors have been identified, including age, gender, genetics, diet, and environmental exposure [2-5].

Cardiovascular diseases (CVDs) are the most common cause of mortality among patients with RA due to accelerated atherosclerosis. It is responsible for approximately one-third to one-half of all deaths related to RA. The risk of CVD among RA patients is estimated to be 50-70% higher compared to the general population. RA patients have twice the risk of developing myocardial infarction (MI) compared to the general population. Reports had previously suggested that dyslipidemia has a pathogenic role in the development and progression of autoimmune disease [6-10].

The lipid profile among RA patients during active or inadequately treated disease is characterized by a decrease in high-density lipoprotein (HDL) levels, whereas for total cholesterol (TC) and low-density lipoprotein (LDL), there have been conflicting reports. Due to the low HDL, there is an increase in the TC-to-HDL ratio, which is an atherogenic index that can be used as a prognostic marker for CVD [11]. An important metabolic feature of RA is catabolism, where the accelerated loss of skeletal muscle takes place, a feature called rheumatoid cachexia that involves the activation of multiple inflammatory mediators such as tumor necrosis factor-alpha (TNF-α), leading to the lowering of TC and HDL [12]. Other inflammatory mediators and cytokines involved in the development of RA include interleukin-1 (IL-1), interleukin-6 (IL-6), interleukin-10 (IL-10), granulocyte-macrophage colony-stimulating factor (GM-CSF), growth hormone, interferon (IFN), and fibroblast growth factor-2 (FGF-2) [13]. These mediators activate both the innate and adaptive immune responses, leading to the production and release of autoantibodies such as rheumatoid factor (RF) and anti-citrullinate protein antibodies (ACPA). The recruitment of the T cells, B cells, and inflammatory cells like macrophages causes the further release of more cytokines and metalloproteases, leading to joint destruction [14].

Treatment with disease-modifying antirheumatic drugs (DMARDs) can influence lipid levels and reduce cardiovascular risk primarily by controlling systemic inflammation. By targeting specific inflammatory pathways and reducing pro-inflammatory cytokines, DMARD therapy may lead to improvements in lipid profiles, including increases in HDL cholesterol and changes in LDL and TC levels. Autoimmune thyroid disease, particularly hypothyroidism, has been reported to occur more frequently among patients with RA, which may further influence lipid metabolism. Moreover, these medications have different effects on the metabolism of lipids. For example, anti-TNF medications, e.g., etanercept, showed a significant increase in HDL levels, while IL-6 inhibitors, e.g., tocilizumab, showed a significant increase in TC and triglycerides. These changes indicate that different types of biological therapies can impact lipid metabolism differently, affecting the overall lipid profile, but overall effective control of RA could reduce the cardiovascular risk of the disease, as evidenced by the correlation between the lipid parameters and inflammatory markers and disease activity level measures by the Disease Activity Score in 28 joints (DAS-28) [15].

Given the significant burden of RA and its complications and the limited data on the prevalence of dyslipidemia among RA patients in the UAE, this study assessed the prevalence of dyslipidemia among RA patients in the UAE and its association with demographic and laboratory variables. The findings are expected to provide valuable insights to aid healthcare professionals in managing dyslipidemia in this population. 

## Materials and methods

This was a single-center retrospective cohort study conducted at the Rheumatology Department of Dubai Hospital, Dubai Health, Dubai, UAE. The diagnosis of RA was confirmed using the 2010 American College of Rheumatology (ACR)/European League Against Rheumatism (EULAR) classification criteria as documented in the patients' medical records. The inclusion criteria were all adults in the UAE from any nationality and both genders aged 21-65 years old and newly diagnosed patients with RA from January 1, 2019, to December 31, 2020. In contrast, the exclusion criteria were patients not in the selected age range, patients diagnosed with RA in a different time period, patients without an RA diagnosis, patients who lost their follow-up visits, or patients who had incomplete laboratory investigations. Patients with incomplete laboratory data for any of the specified parameters were excluded to ensure consistency across all three time points. Thyroid-stimulating hormone (TSH) was included to identify thyroid dysfunction, which may influence lipid metabolism, while liver function tests (LFTs) were included to assess for metabolic comorbidities such as fatty liver disease that could affect lipid profiles. Data were retrospectively collected from electronic medical records between March 10, 2024, and April 5, 2024, covering laboratory and demographic parameters from two years before and two years after diagnosis for the patients who met the inclusion criteria. Two investigators conducted this process, and all the entries were double-checked to minimize errors. Any discrepancies were sorted by consulting a third investigator. Data collected included patient demographics and medical history (medical record number, age, gender, nationality, BMI, and date of RA diagnosis). Laboratory investigations included lipid profile, white blood cells (WBC), TSH, LFT, creatinine, erythrocyte sedimentation rate (ESR), and C-reactive protein (CRP). For each patient, lipid profile data were collected retrospectively from the medical records at three time points: up to two years before the date of RA diagnosis, at the time of diagnosis, and up to two years after diagnosis. Patients with missing data or incomplete data were excluded from the study. Dyslipidemia was defined based on the reference ranges used in Dubai Hospital's laboratory following the 2019 European Society of Cardiology (ESC)/European Atherosclerosis Society (EAS) guidelines for the management of dyslipidemia: TC (≥190 mg/dL), triglycerides (≥150 mg/dL), LDL (≥115 mg/dL), or HDL (<48 mg/dL) [16]. The study was approved by the University Students and Resident Research Subcommittee 1 (approval number: MBRU IRB-2023-78). 

Statistical analysis

Data analysis was done using IBM SPSS Statistics for Windows, Version 26.0 (IBM Corp., Armonk, New York, United States). Numerical variables were presented as mean±standard deviation or median with interquartile ranges, as appropriate. Categorical variables were presented as counts and percentages. Fisher's exact test was used for small sample sizes in categorical variables, while McNemar's test was used to analyze paired categorical data (p<0.05).

## Results

The study included a total of 104 RA patients reporting to Dubai Hospital during the period from January 1, 2019, to December 31, 2020. The cohort was predominantly female (88, 84.6%), with a mean age of 49, predominantly middle-aged (73, 70.2%), and predominantly Emirati (76, 73.1%). The mean BMI was 30.9(±7.2), with more than half of the patients classified as obese (55, 52.9%). Demographic and baseline characteristics of the included sample are presented in Table 1. 

**Table 1:** Demographics and baseline characteristics of the included sample

Characteristic	n (%)
Age group	
19-39 years (young adults)	21 (20.2%)
40-64 years (middle-aged)	73 (70.2%)
≥65 years (elderly)	10 (9.6%)
Gender	
Female	88 (84.6%)
Male	16 (15.4%)
Nationality	
Emirati	76 (73.1%)
Non-Emirati	28 (26.9%)
BMI category	
Normal	22 (21.2%)
Overweight	27 (26%)
Obese	55 (52.9%)

Two years before the diagnosis of RA, 78 (75%) patients had dyslipidemia, while 26 (25%) had normal lipid profiles. Most of the patients during that period exhibited high LDL levels (55, 52.9%), followed by high TC (52, 50%), low HDL (41, 39.4%), and high triglyceride (17, 16.3%). At the time of RA diagnosis, the prevalence of dyslipidemia was 76 (73.1%). Many of the patients during this period had low HDL (52, 50%). At the time of RA diagnosis, both TC and LDL were high in 44 (42.3%) patients, and triglyceride was high in 16 (15.4%) patients. Two years after the diagnosis of RA, the prevalence of dyslipidemia decreased slightly to 72 (69.2%), while 32 (30.8%) had normal lipid profiles. The level of elevated LDL remained the same at 44 (42.3%). After RA diagnosis, both TC and low HDL were abnormal in 41 (39.4%) patients, and high triglyceride decreased slightly to 13 (12.5%) (Tables 2-3).

**Table 2:** Dyslipidemia prevalence at different time points RA: rheumatoid arthritis

Time point	Dyslipidemia present, n (%)	Dyslipidemia absent, n (%)
Before RA diagnosis	78 (75%)	26 (25%)
At the time of RA diagnosis	76 (73.1%)	28 (26.9%)
After RA diagnosis	72 (69.2%)	32 (30.8%)

**Table 3:** Lipid profiles over time (mean±SD) RA: rheumatoid arthritis

Lipid parameter	Before RA diagnosis	At the time of RA diagnosis	After RA diagnosis
Total cholesterol	188.02±42.36	183.88±39.53	185.39±41.24
Triglycerides	106.71±45.18	105.77±45.50	101.52±41.36
Low-density lipoprotein	118.05±37.26	112.57±37.39	110.78±35.73
High-density lipoprotein	53.36±12.77	52.08±13.37	54.67±14.85

McNemar's test was used to evaluate the change in the dyslipidemia trend before and after RA diagnosis within the same patients. The results showed no significant difference (p=0.329). Of the selected participants, 58 (55.8%) had dyslipidemia before and after RA diagnosis, while 12 (11.5%) had normal lipid profiles the whole time. Only 20 subjects (19.2%) who had dyslipidemia before RA diagnosis normalized their lipid profile after RA diagnosis, while 14 (13.5%) only developed dyslipidemia after RA diagnosis. 

To assess the difference in the prevalence of dyslipidemia in the study sample across gender, age, and nationality subgroups, Fisher's exact test was used. Among females, before RA diagnosis, dyslipidemia was reported in 65 subjects (73.9%) (p=0.755), persisted at the time of RA diagnosis (65, 73.9%) (p=0.761), and decreased mildly (61, 69.3%) after RA diagnosis (p=1.000), while, among males, before RA diagnosis, 13 (81.3%) (p=0.755) had dyslipidemia which decreased to 11 (68.8%) at the time of RA diagnosis (p=0.761) and persisted (11, 68.8%) after RA diagnosis (p=1.000). Across age groups, dyslipidemia was most prevalent among middle-aged patients (59, 80.8%), followed by young adults (12, 57.1%) and the elderly (7, 70%) before RA diagnosis, with no significant difference (p=0.081). At the time of RA diagnosis, dyslipidemia prevalence was 74% among middle-aged patients, 76.2% among young adults, and 60% among elderly patients, with no statistically significant difference observed between age groups (p=0.606).** **Moreover, after RA diagnosis, dyslipidemia was still seen in middle-aged patients (53, 72.6%), young adults (14, 66.7%), and the elderly (5, 50%), but without a significant difference (p=0.334). Dyslipidemia prevalence was higher in Emirati patients before RA diagnosis (55, 72.4%) (p=0.444) and persisted at the time of RA diagnosis (55, 72.4%) (p=1.000) and slightly decreased to 51 (67.1%) after RA diagnosis (p=0.483), while the dyslipidemia prevalence in non-Emirati patients before RA was 23 (75%) (p=0.444) and slightly decreased to 21 (75%) at the time of RA diagnosis (p=1.000) and persisted (21, 75%) after RA diagnosis. There was no significant difference (p=0.483) (Table 4).

**Table 4:** Subgroup analysis of dyslipidemia prevalence after RA diagnosis RA: rheumatoid arthritis

Subgroup	Dyslipidemia present, n (%)	Dyslipidemia absent, n (%)	P-value (Fisher's exact test)	
Gender				
Female	61 (69.3%)	27 (30.7%)		1.000
Male	11 (68.8%)	5 (31.3%)		
Age group				
19-39 years	14 (66.7%)	7 (33.3%)	0.334	
40-64 years	53 (72.6%)	20 (27.4%)		
≥65 years	5 (50%)	5 (50%)		
Nationality				
Emirati	51 (67.1%)	25 (32.9%)		0.483
Non-Emirati	21 (75%)	7 (25%)		

Other laboratory findings revealed that TSH levels before RA diagnosis were elevated in three (2.9%) and increased to eight (7.7%) after RA diagnosis. Inflammatory markers that were recorded included ESR, which was found abnormally elevated in 82 (78.8%) patients before RA diagnosis and decreased slightly to 71 (68.3%) after RA diagnosis, while CRP level was found abnormally elevated in 36 (34.6%) patients before RA diagnosis and increased mildly to 44 (42.3%) after RA diagnosis (Table 5). Statistical testing of additional laboratory findings (e.g., ESR, CRP, TSH) was not performed due to sample size constraints and because only lipid-related parameters were designated as the primary outcomes of this study.

**Table 5:** Other laboratory findings in RA patients RA: rheumatoid arthritis; WBC: white blood cells; TSH: thyroid-stimulating hormone; LFT: liver function test; ESR: erythrocyte sedimentation rate; CRP: C-reactive protein

Laboratory investigation	Before RA diagnosis n (%)	After RA diagnosis n (%)
WBC	Low: 3 (2.9%)	Low: 6 (5.8%)
	Normal: 99 (95.2%)	Normal: 92 (88.5%)
	High: 2 (1.9%)	High: 6 (5.8%)
TSH	Low: 1 (1%)	Low: 1 (1%)
	Normal: 100 (96.2%)	Normal: 95 (91.3%)
	High: 3 (2.9%)	High: 8 (7.7%)
Creatinine	Low: 4 (3.8%)	Low: 2 (1.9%)
	Normal: 87 (83.7%)	Normal: 90 (86.5%)
	High: 13 (12.5%)	High: 12 (11.5%)
LFT	Normal: 94 (90.4%)	Normal: 87 (83.7%)
	Abnormal: 10 (9.6%)	Abnormal: 17 (16.3%)
ESR	Normal: 22 (21.2%)	Normal: 33 (31.7%)
	Abnormal: 82 (78.8%)	Abnormal: 71 (68.3%)
CRP	Normal: 68 (65.3%)	Normal: 60 (57.7%)
	Abnormal: 36 (34.6%)	Abnormal: 44 (42.3%)

## Discussion

This study contributes novel data from the UAE, a region where the prevalence and lipid profile patterns of RA remain largely understudied. The study sample included predominantly female, middle-aged, and obese participants, which aligns with known epidemiological trends in autoimmune diseases. RA is reported to be two to three times more common in females than in males [17], and its incidence is four to five times higher among middle-aged adults compared to other age groups [18]. Also, the majority were obese patients (52.9%). This is consistent with previous studies that identified obesity as a modifiable risk factor that exerts its risk at an early asymptomatic phase of RA development. It is known that adipose tissue can secrete pro-inflammatory cytokines. It is possible that pro-inflammatory cytokines secreted by adipose tissue contribute to the development of an autoimmune response before the onset of RA clinical symptoms [19]. Moreover, the study included 76 (73.1%) Emirati patients and 28 (26.9%) non-Emirati patients, possibly due to the nature of the study setting, classified as a governmental healthcare entity. 

Dyslipidemia was mostly reported in the two years before RA diagnosis (75%) with predominantly high levels of LDL (52.9%), followed by a high level of TC (50%), a low level of HDL (39.4%), and high triglyceride (16.3%). In contrast to these findings, a study by Myasoedova et al. [20] reported a significant decrease in LDL and TC five years before RA diagnosis, while HDL and triglyceride levels remained the same before and after RA diagnosis. The difference might be explained by the difference in follow-up duration. In our sample, 65 (73.9%) female patients had dyslipidemia before RA diagnosis, continued to have dyslipidemia at the time of RA diagnosis, and only showed a mild decrease, possibly due to treatment, where 61 (69.3%) persisted in having dyslipidemia after RA diagnosis. A study by Turesson et al. [21] indicated that compared to men, women had a higher level of TC before developing RA, suggesting that high TC could be a risk factor for developing RA in women, that sex-specific exposures may modify the impact of lipid on the risk of RA, and that hormone-related metabolic pathways may contribute to the development of RA. During this time, inflammatory markers were elevated with high ESR in 82 (78.8%) patients, and CRP was elevated in 36 (34.6%) patients.

At the time of RA diagnosis, dyslipidemia was still recorded in 76 (73.1%) patients with predominantly low HDL levels (52, 50%), followed by an equal number of high TC and LDL (44, 42.3%) and high triglyceride (16, 15.4%), respectively. These findings agree with the lipid paradox, where the levels of TC, LDL, and HDL decrease under hyperinflammatory conditions despite having an increased risk of CVD. During systemic inflammation in RA, pro-inflammatory cytokines, mainly IL-6, IL-1, and TNF-α, show a significant increase, leading to an alteration in lipid profile and a decrease in LDL levels. These changes are due to increased oxidation, increased endothelial deposition, and degradation. Additionally, low HDL can be due to HDL catabolism that increases with inflammation, the reduction of HDL cholesterol efflux capacity, and the HDL particles becoming pro-atherogenic, leading to weakened anti-atherosclerosis function, which in turn can increase LDL oxidation and the risk of CVD [22,23].

After RA diagnosis, dyslipidemia persisted in 69.2% of the patients with persistent high LDL (44, 42.3%), followed by an equal number of high TC and low HDL (41, 39.4%) and high triglyceride (13, 12.5%). Dyslipidemia was highest among the middle-aged group (53, 72.6%). These findings contradict other studies reporting a significant reduction in dyslipidemia after initiating RA treatment. These variations might be explained by the differences in the treatment regimen, population characteristics, and study design. Similar to our findings, Kholmirzayev [24], who compared three different groups with different treatment regimens in patients with RA, reported that high RA disease activity was associated with high CRP and dyslipidemia and the severity of dyslipidemia impairment can be correlated to increased RA severity. The study also demonstrated improved lipid profile and reduced RA activity after six months and a year of integrating statin (rosuvastatin) into the regimen compared with the others. In our sample, during this period, the inflammatory markers ESR and CRP were elevated in 71 (68.3%) and 44 (42.3%) patients, respectively.

The lipid profile was consistently variable in most subjects throughout the whole follow-up period in the current sample in all parameters. Notably, 58 (55.8%) had dyslipidemia throughout the whole study period, while 12 (11.5%) never had dyslipidemia throughout the study. Also, 20 (19.2%) patients had dyslipidemia before RA diagnosis and then afterward had normal lipid profiles, which could be due to the treatment, while 14 (13.5%) developed dyslipidemia for the first time after RA diagnosis, which could be due to the course of the illness [25]. Moreover, 50% of the patients had abnormal HDL levels at the time of RA diagnosis, which also persisted afterward. Triglyceride was predominantly normal all the time, with abnormalities seen only in 12.5-16.3% of the patients.

Autoimmune thyroid disease, particularly hypothyroidism, has been reported to have a bidirectional association with RA. In the current study, elevated TSH levels were observed in three patients (2.9%) before RA diagnosis and increased to eight patients (7.7%) after diagnosis. These findings highlight the importance of monitoring RA patients for comorbid autoimmune conditions, such as hypothyroidism, in addition to CVD. According to EULAR, while there is no official guideline that recommends testing TSH in all RA patients, expert recommendations suggest testing at the time of RA diagnosis and then periodically or if the patient is exhibiting symptoms of hypothyroidism and has been on a long course of methotrexate or glucocorticoids [26-28].

This study had some limitations, including being a single-center study with a short time frame and a small sample size, which might limit the generalizability of the findings. The design did not take into consideration the seropositivity status (RF and ACPA) that might impact the lipid profile. Disease activity assessed using DAS-28 may have influenced lipid profile changes and inflammatory marker trends. Additionally, the potential effects of corticosteroid therapy and lipid-lowering medications on lipid levels could not be analyzed due to data limitations and the retrospective study design. Future studies with larger sample sizes incorporating disease activity measures, autoantibody status, and detailed medication data are needed to better characterize dyslipidemia in RA.

## Conclusions

Dyslipidemia has a high prevalence among RA patients in Dubai, with many patients having persistent lipid abnormalities before and after RA diagnosis. This study highlights the need for comprehensive cardiovascular risk assessment and routine lipid profile monitoring in patients with RA. Early identification and management of dyslipidemia can improve long-term cardiovascular outcomes in this population. Early interventions with lifestyle modification or lipid-lowering medications such as statins should be considered once dyslipidemia is diagnosed to reduce cardiovascular risk in this population. Future studies should focus on studying longitudinal lipid profile changes in RA patients at different treatment regimens and exploring the trends of the lipid profile and outcome in patients with strict control of RA on cardiovascular risk.
